# First reported case of a longevity overestimation error in the new Medtronic tablet‐based device programmer

**DOI:** 10.1002/joa3.12406

**Published:** 2020-07-16

**Authors:** Pedram Kazemian, Lei Xu

**Affiliations:** ^1^ Deborah Heart and Lung Center Lawrenceville NJ USA

**Keywords:** Covid19, device interrogation, longevity overestimation, programmer error, tablet‐based programmer

## Abstract

With the advancement and miniaturization of mobile technologies, major device companies are replacing the traditional cardiac rhythm device programmers with smaller and more efficient tablet‐based systems. As clinicians rely on data obtained from multitude of these systems, it is imperative that they provide consistent, reliable, and reproducible results. In this case report, we illustrate, for the first time, a major discrepancy between remote monitoring data, a conventional device programmer, and the new tablet‐based Medtronic CareLink SmartSync Device Manager which erroneously overestimated the battery longevity in a pacemaker‐dependent patient whose device had reached Recommended Replacement Time (RRT) status.

## Graphical abstract

This is a first reported case of overestimation error in battery longevity by the new Medtronic tablet‐based programmer in a pacemaker patient.

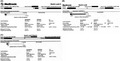

## 1 INTRODUCTION

Interrogations of cardiac implantable electronic devices (CIEDs) using traditional device programmers have been the cornerstone of device monitoring and follow‐up. Widespread adoption of remote monitoring has brought additional safety and convenience. In cases of national emergencies such as covid‐19 virus pandemic, remote monitoring of CIEDs could safely substitute the majority of in‐person visits whereby reducing the spread of the infection, a policy that we have adopted in our institution.

With the widespread availability of mobile technologies, there is a concerted effort by most major device manufacturers to replace the traditional cardiac device programmers with mobile platforms. Medtronic (MDT) CareLink SmartSync™ is the next‐generation tablet‐based device manager that uses BlueSync™ technology for wireless connectivity and functions as a portable device programmer and pacing system analyzer to replace the older Medtronic CareLink programmers.^1^

Here, we report a case of a pacemaker‐dependent patient whose MDT CRT‐P device was identified to have reached RRT status according to the remote monitoring but interrogation of his device by the new CareLink SmartSync™ displayed a remaining longevity of 4 years.

## 2 CASE REPORT

**Figure 1:**
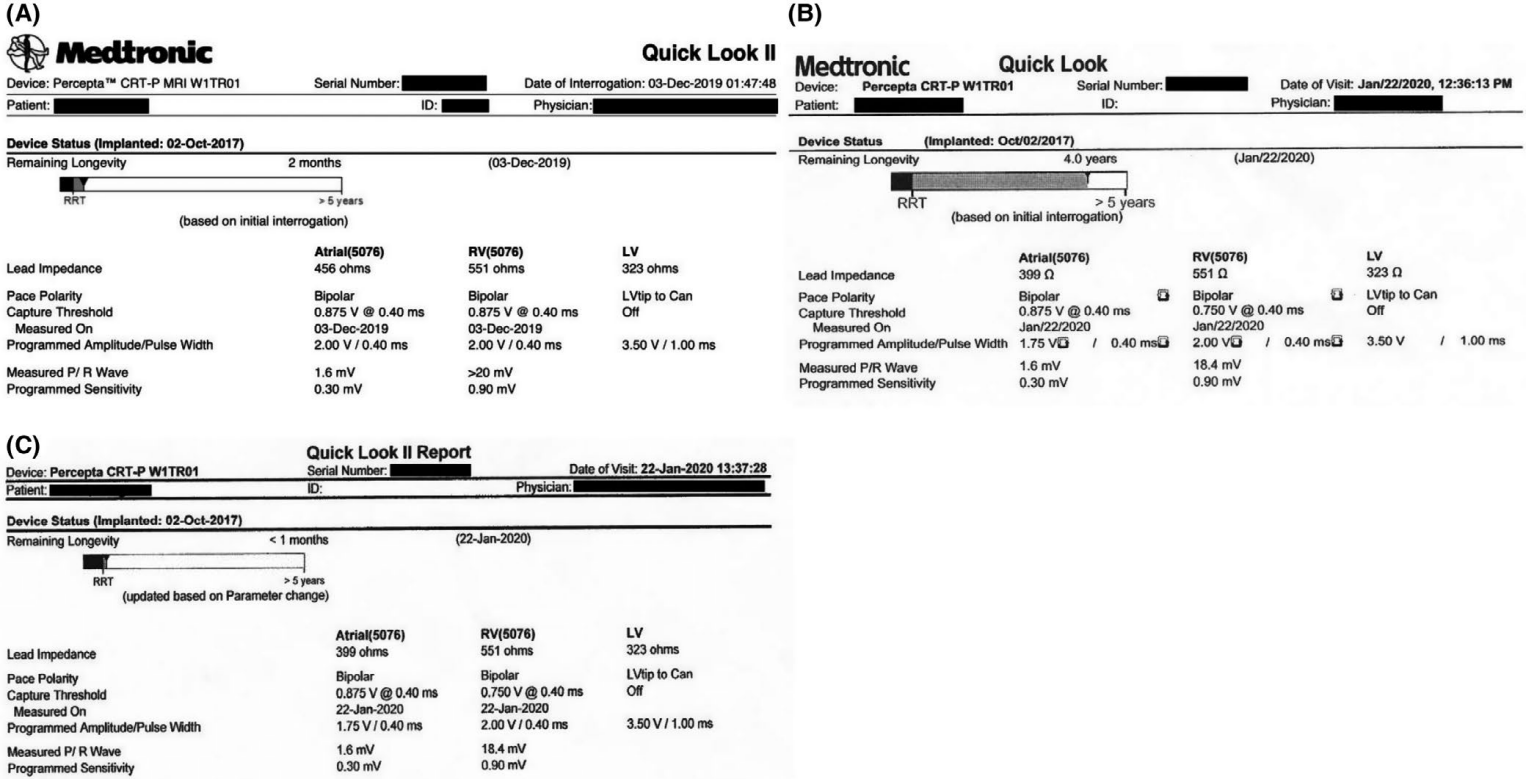
A, Remote monitoring of device on Dec 3^rd^, 2019, demonstrated the device to be at RRT with projected battery longevity of 2 months. B, Device interrogation using tablet‐based CareLink™ SmartSync™ Device Manager on January 22^nd^, 2020, displayed a remaining longevity of 4 years. C, Device interrogation with a conventional Model 2090 programmer on the same day showed the remaining longevity was 1 month

A CareLink transmission of a MDT CRT‐P device (Percepta™ CRT‐P MRI SureScan™) on December 3, 2019 had shown a longevity of 2 months and battery voltage of 2.64V. This device belonged to an 82‐year‐old female with complete heart block and history of paroxysmal atrial fibrillation which was originally implanted in October of 2017. Patient was pacemaker‐dependent with no underlying atrioventricular conduction and biventricular pacing burden of 99.5%. (Figure 1A, Figure S1).

She was scheduled to have a generator replacement procedure on January 22nd, 2020. However, on the day of her procedure, her device interrogation using CareLink™ SmartSync™ Device Manager (Percepta Application Model Number D00U004) displayed a remaining longevity of 4 years and battery voltage of 2.61 V. (Figure 1B, Figure S1). As patient was pacemaker dependent and the battery voltage of 2.61 V was inconsistent with the projected longevity, an older conventional device programmer (CareLink™ 2090) was used to determine the exact device battery status. When the device was interrogated with a Model 2090 programmer, the remaining longevity was 1 month with a battery voltage of 2.61 V. (Figure 1C, Figure S1).

Given the clinical status of the patient, consistent low battery voltage readings, and despite the projected longevity of the table‐based programmer, a decision was made to proceed with device generator change which was performed without any complications.

The case was reported to the MDT company for assessment. After analyzing the data, on February 7th, 2020, MDT confirmed the potential for the Remaining Longevity Estimate to be inaccurate (overestimated) when the device approaches RRT indicator. This overestimation error only occurs when the device is interrogated with a SmartSync Device Manager and the device is within approximately 180 days of its Recommended Replacement Time (RRT) indicator. This error does not occur with older programmers including Model 2090, Encore, or with CareLink session. Also, other devices supported by the SmartSync Device Manager are not affected by this error according to the MDT report.

The root cause was attributed to an error in the rewriting of Percepta application software from the Model 2090 programmer to the SmartSync platform (application model number D00U004). They further acknowledged this to be the first reported occurrence of this behavior, and pledged to correct this error by a software update in a future release of the SmartSync application. Until this software error is corrected, this issue could recur.

## 3 DISCUSSION

This case illustrates a potentially fatal error in the new tablet‐based MDT CareLink™ SmartSync™ Device Manager resulting in overestimation of battery longevity when the device is nearing RRT indicator.

In the last 2 years, a few software issues related to MDT programmers as well as remote monitoring applications have been identified.

In 2018, cybersecurity vulnerabilities associated with the internet connection of Medtronic programmers (models 2090 and 29901) were discovered.^2^, ^3^ In 2019, MDT identified the potential for Medtronic programmers, including 2090 CareLink™, 29901 Encore™, and CareLink SmartSync™ Device Manager as well as remote monitoring software applications to display an inaccurate remaining longevity estimate for a subset of implanted cardiac device models. However, in the latter case, RRT remained accurate and no clinical adverse events were reported as the inaccuracy occurred in the middle phase of the device life.^4^

These experiences highlight the importance of exercising caution when interpreting the results of device interrogations using Medtronic programmers especially in pacemaker‐dependent patients. A few practical strategies to detect such errors include: (a) identifying discrepancies between device battery voltage and the estimated longevity; (b) confirmation of the device status using different programmers and remote monitoring data when there is suspicion of erroneous report; and (c) until further update of the tablet‐based programmer, conventional device programmer (CareLink™ 2090), or remote monitoring should be used for interrogation of CRT‐P devices.

Finally, as new technologies are developed for monitoring CIDEs, it is important to be cognizant of potential errors associated with any single software‐based system and to exercise caution when the reports are not clinically consistent.

## CONFLICT OF INTERESTS

The authors declare no conflict of interests for this article.

## Supporting information

Fig S1
