# Supplementary material for: First reported case of a longevity overestimation error in the new Medtronic tablet‐based device programmer
Source: J Arrhythm. 2020 Jul 16;36(5):929–31. doi: 10.1002/joa3.12406 (PMC7532279; doi:10.1002/joa3.12406)
Supplement: Supplementary file 1 [file JOA3-36-929-s001.docx]

A

Medtronic — Quick Look II

Device: Percepta™ CRT-P MRI W1TR01	Serial Number: [redacted]	Date of Interrogation: 03-Dec-2019 01:47:48
Patient: [redacted]	ID: 145324	Physician: [redacted]

Device Status (Implanted: 02-Oct-2017)

Remaining Longevity	2 months	(03-Dec-2019)

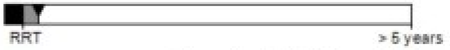

(based on initial interrogation)

	Atrial(5076)	RV(5076)	LV
Lead Impedance	456 ohms	551 ohms	323 ohms
Pace Polarity	Bipolar	Bipolar	LVtip to Can
Capture Threshold	0.875 V @ 0.40 ms	0.875 V @ 0.40 ms	Off
Measured On	03-Dec-2019	03-Dec-2019	
Programmed Amplitude/Pulse Width	2.00 V / 0.40 ms	2.00 V / 0.40 ms	3.50 V / 1.00 ms
Measured P/ R Wave	1.6 mV	>20 mV	
Programmed Sensitivity	0.30 mV	0.90 mV	

Parameter Summary

Mode	DDDR	Lower Rate	70 bpm	AdaptivCRT	Nonadaptive CRT
Mode Switch	171 bpm	Upper Track	130 bpm	V. Pacing	LV->RV
		Upper Sensor	120 bpm	Paced AV	130 ms
				Sensed AV	100 ms

Detection		Rates	Therapies
AT/AF	Monitor	>171 bpm	All Rx Off
VT	Monitor	>150 bpm	

Clinical Status	Since 03-Sep-2019
**Treated**	
AT/AF(Monitor)	
**Monitored**	
VT (>4 beats)	3
Fast A&V	0
AT/AF	7
Time in AT/AF	<0.1 hr/day (<0.1%)
**Functional**	**Last Week**
Patient Activity	3.6 hr/day

Cardiac Compass Trends (Oct-2018 to Dec-2019)

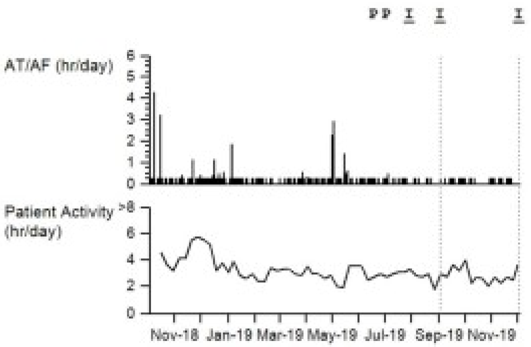

Therapy Summary	AT/AF	Pacing	(% of Time Since 03-Sep-2019)
Pace-Terminated Episodes	0	VP	99.4% (MVP Off)
		Effective	99.4%
		AP	44.5%

OBSERVATIONS (1)

- RV Capture Management: Actual safety margin (2.3 X) > programmed margin (2.0 X).

Copyright © 2001-2019 Medtronic, Inc

B

Medtronic Quick Look

Device: **Percepta CRT-P W1TR01** Serial Number: [redacted] Date of Visit: **Jan/22/2020, 12:36:13 PM**
Patient: [redacted] ID: Physician: [redacted]

Device Status **(Implanted: Oct/02/2017)**

Remaining Longevity 4.0 years (Jan/22/2020)

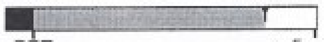

(based on initial interrogation)

	**Atrial(5076)**	**RV(5076)**	**LV**
Lead Impedance	399 Ω	551 Ω	323 Ω
Pace Polarity	Bipolar	Bipolar	LVtip to Can
Capture Threshold	0.875 V @ 0.40 ms	0.750 V @ 0.40 ms	Off
Measured On	Jan/22/2020	Jan/22/2020	
Programmed Amplitude/Pulse Width	1.75 V / 0.40 ms	2.00 V / 0.40 ms	3.50 V / 1.00 ms
Measured P/R Wave	1.6 mV	18.4 mV	
Programmed Sensitivity	0.30 mV	0.90 mV	

Parameter Summary

Mode	DDD	Lower Rate	60 bpm	AdaptivCRT	Nonadaptive CRT
Mode Switch	171 bpm	Upper Track	130 bpm	V. Pacing	LV->RV
		Upper Sensor	120 bpm	Paced AV	130 ms
				Sensed AV	100 ms

**Detection**		**Rates**	**Therapies**
AT/AF	Monitor	>171 bpm	All Rx Off
VT	Monitor	>150 bpm	

Clinical Status **Since Jun/28/2019**

Treated
AT/AF(Monitor)

**Monitored**		
VT(>4 beats)		4
Fast A&V		0
AT/AF		19
Time in AT/AF	<0.1 h/d	(<0.1 %)
**Functional**		**Last Week**
Patient Activity		2.6 h/d

Cardiac Compass Trends (Nov/2018 to Jan/2020)

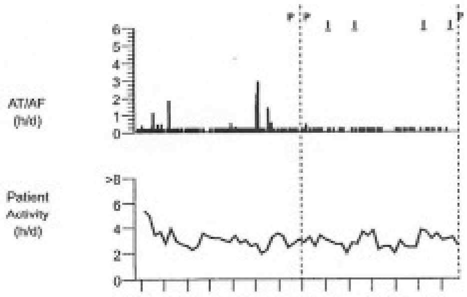

**Therapy Summary**	**AT/AF**
Pace-Terminated Episodes	0

**Pacing**	**(% of Time Since Jun/28/2019)**	
VP	99.5 %	(MVP Off)
Effective	99.5 %	
AP	43.2 %	

© 2017 Medtronic

C

Quick Look II Report

Device: **Percepta CRT-P W1TR01** Serial Number: [redacted] Date of Visit: **22-Jan-2020 13:37:28**

Patient: [redacted] ID: Physician: [redacted]

Device Status (Implanted: 02-Oct-2017)

Remaining Longevity < 1 months (22-Jan-2020)

RRT > 5 years

(updated based on Parameter change)

	Atrial(5076)	RV(5076)	LV
Lead Impedance	399 ohms	551 ohms	323 ohms
Pace Polarity	Bipolar	Bipolar	LVtip to Can
Capture Threshold	0.875 V @ 0.40 ms	0.750 V @ 0.40 ms	Off
Measured On	22-Jan-2020	22-Jan-2020	
Programmed Amplitude/Pulse Width	1.75 V / 0.40 ms	2.00 V / 0.40 ms	3.50 V / 1.00 ms
Measured P/ R Wave	1.6 mV	18.4 mV	
Programmed Sensitivity	0.30 mV	0.90 mV	

Parameter Summary

Mode	DDD	Lower Rate	60 bpm	AdaptivCRT	Nonadaptive CRT
Mode Switch	171 bpm	Upper Track	130 bpm	V. Pacing	LV->RV
		Upper Sensor	120 bpm	Paced AV	130 ms
				Sensed AV	100 ms

**Detection**		**Rates**	**Therapies**
AT/AF	Monitor	>171 bpm	All Rx Off
VT	Monitor	>150 bpm	

Clinical Status **Since 28-Jun-2019**

Treated

AT/AF(Monitor)

Monitored

VT (>4 beats)	4
Fast A&V	0
AT/AF	19
Time in AT/AF	<0.1 hr/day (<0.1%)

**Functional**	**Last Week**
Patient Activity	2.6 hr/day

Cardiac Compass Trends (Nov-2018 to Jan-2020)

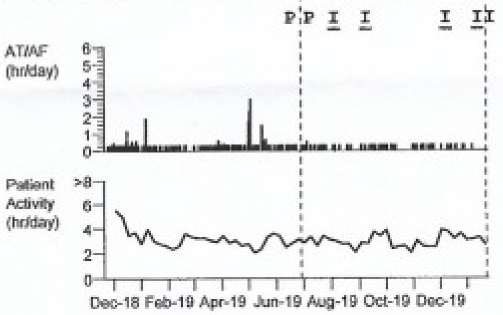

**Therapy Summary**	**AT/AF**
Pace-Terminated Episodes	0

**Pacing**	**(% of Time Since 28-Jun-2019)**
VP	99.5 % (MVP Off)
Effective	99.5 %
AP	43.2 %

OBSERVATIONS (2)

- Possible OptiVol fluid accumulation: 08-Sep-2019 -- 10-Sep-2019.
- RV Capture Management: Actual safety margin (2.7 X) > programmed margin (2.0 X).
